# Correlates of Level and Loss of Grip Strength in Later Life: Findings from the English Longitudinal Study of Ageing and the Hertfordshire Cohort Study

**DOI:** 10.1007/s00223-017-0337-5

**Published:** 2017-10-22

**Authors:** H. E. Syddall, L. D. Westbury, S. C. Shaw, E. M. Dennison, C. Cooper, C. R. Gale

**Affiliations:** 1 MRC Lifecourse Epidemiology Unit, Southampton General Hospital, University of Southampton, Southampton, SO16 6YD UK; 2 Victoria University of Wellington, Wellington, New Zealand; 3 NIHR Southampton Biomedical Research Centre, University of Southampton and University Hospital Southampton NHS Foundation Trust, Southampton, UK; 4 NIHR Musculoskeletal Biomedical Research Unit, University of Oxford, Oxford, UK; 5 Department of Psychology, Centre for Cognitive Ageing and Cognitive Epidemiology, University of Edinburgh, Edinburgh, UK

**Keywords:** Grip strength, Involutional decline, Risk factors, Later life

## Abstract

Characterisation of grip strength (GS) using isometric dynamometry is central to the definition of sarcopenia. Determinants of low GS include: older age, shorter stature, low physical activity, poor nutrition, socioeconomic disadvantage and multimorbidity. Less is known about risk factors for accelerated loss of GS. We investigated determinants of level and 8-year loss of GS in 3703 men and women (aged 52–82 years) in the English Longitudinal Study of Ageing (ELSA). Four hundred and forty-one men and women (aged 59–71 years) who participated in a 10-year follow-up of the Hertfordshire Cohort Study (HCS) were used for replication. Variables were harmonised between cohorts. Change in GS was characterised using mixed-effects models in ELSA and a residual change approach in HCS and analysed for men and women combined. Men in ELSA and HCS had higher average levels of GS at baseline, and accelerated rates of loss, compared with women. In ELSA, older age, shorter stature and multimorbidity were correlated with lower level, and accelerated rate of loss, of GS in both sexes (accelerated loss of 0.04 (95% CI 0.00–0.08) standard deviation scores per additional morbidity after multivariable adjustment). Socioeconomic disadvantage, low level of physical activity and poorer self-reported health were also correlated with low GS level, but not loss rate, after multivariable adjustment. Analysis in HCS yielded similar results. Our results identify multimorbidity as a modifiable determinant of loss of muscle strength in later life, and raise the possibility that developmental influences may impact on rate of involutional decline in muscle strength.

**Electronic supplementary material:**

The online version of this article (doi:10.1007/s00223-017-0337-5) contains supplementary material, which is available to authorised users.

## Introduction

Sarcopenia is an age-related syndrome characterised by aggressive and general loss of skeletal muscle mass and strength [1]. It is a major contributor to the risk of physical frailty, functional impairment, poor health-related quality of life and premature death [2]. Sarcopenia has recently been recognised as a specific disease by assignment of a single code within the International Classification of Diseases [3]. It is responsible for considerable health care expenditure. Annual direct medical costs attributable to the disorder have been estimated at around $20 billion in the United States in 2000 [4].

Characterisation of muscle strength using isometric dynamometry is central to the definition of sarcopenia [1]. Epidemiological studies typically assess muscle strength using isometric hand grip, and reference ranges for grip strength throughout the life course have been determined in the UK [5] and elsewhere [6, 7]. Grip strength in later life depends upon the peak grip strength attained during growth and young adulthood, as well as the subsequent rate of loss. In other musculoskeletal tissues, for example the skeleton, differential determinants of peak bone mass and rate of bone loss have been observed [8–11].

Determinants of low grip strength level include older age [5], shorter stature [12], poor nutrition [13], low physical activity [14], socioeconomic disadvantage [15, 16] and multimorbidity [17]. There has been much less research into risk factors for accelerated loss of grip strength in later life. Several studies have concentrated solely on investigating age and sex differences in grip strength trajectory in older people [7, 18–20]. Most such investigations, though not all [21], have shown that grip strength declines with age in both sexes and that the decline is faster in men. Only a few longitudinal studies have examined the role of a broader range of potential determinants of change in grip strength [22–25], and to date, few consistent predictors of grip strength decline have been identified. In order to establish with greater certainty which factors are predictive of grip strength decline in men and women, there is a need for further, large longitudinal studies of older people of both sexes and a wide range of ages; this study addresses these concerns.

We used data from two well-characterised cohorts of older people, the English Longitudinal Study of Ageing (ELSA) [26] and the Hertfordshire Cohort Study (HCS) [27], to conduct a cross-cohort examination [28, 29] of the determinants of both grip strength level and change in later life.

## Methods

We analysed data from ELSA for our principal analyses and used HCS for replication. We identified sufficiently comparable variables detailing: demographic factors, anthropometry, socioeconomic position, lifestyle risk factors, physical function and morbidity. The cohorts are described below; profiles have been published previously [26, 27].

### The English Longitudinal Study of Ageing

The initial sample for ELSA was based on people aged 50 years and older who had participated in the Health Survey for England in 1998, 1999 or 2001. It was drawn by postcode sector, stratified by health authority and proportion of households in non-manual socioeconomic groups. The initial survey took place in 2002–2003. Subsequent waves of data collection took place at 2 yearly intervals. At 4 yearly intervals, core sample members who completed the main interview are invited to have a visit from a nurse that includes measurements of physical function and anthropometry. Refreshment samples drawn from the Health Survey for England were added at Wave 3 and 4 to maintain the representation of people aged 50–75. The current study uses data from Waves 2, 4 and 6. Ethical approval was obtained from the NHS Multicentre Research Ethics Committee in London. Participants gave written informed consent.

At Wave 2, the following characteristics were ascertained at a nurse-administered home interview (see Online Appendix 1 for full details): marital status, housing tenure, occupational characteristics, smoking status, frequency of alcohol consumption, self-reported physical activity in work and daily life, self-rated health, measured height and weight; and previous diagnosis by a doctor of high blood pressure/hypertension, angina, heart attack, diabetes or high blood sugar, a stroke or osteoporosis. At Waves 2, 4 and 6, participants had grip strength measured three times for each hand using the Smedley dynamometer; the highest grip measurement at each time point was used for analysis. The ELSA analysis sample for this paper comprised 3703 participants with complete grip strength data at the three waves.

### The Hertfordshire Cohort Study

The HCS comprises 1579 men and 1418 women born in Hertfordshire in 1931–1939 and who still lived there in 1998–2004. The following characteristics were ascertained at a nurse-administered home interview (see Online Appendix 1): marital status, housing tenure, current or most recent full time occupation and husband’s details for ever-married women; smoking status, weekly alcohol consumption, customary physical activity level, self-rated health, typical angina according to the Rose chest pain questionnaire and previous diagnosis of high blood pressure, heart attack, diabetes or stroke/transient ischaemic attack. Participants subsequently attended a clinic at which height and weight were measured, a 2-h fasted oral glucose tolerance test (OGTT) was performed using 75 g anhydrous glucose, and resting blood pressure was measured. Grip strength was assessed three times for each hand using a Jamar dynamometer; the highest measurement was used for analysis. Participants also underwent a DXA scan. The HCS analysis sample for this paper comprised 441 participants who had grip strength measured both at baseline and, according to identical protocol [30], during a follow-up study [median follow-up time 10.6 years (inter-quartile range 10.1–11.6)] [31].

The baseline HCS had ethical approval from the Hertfordshire and Bedfordshire Local Research Ethics Committee and the follow-up had ethical approval from the East and North Hertfordshire Ethical Committees. Participants gave written informed consent.

### Statistical Methods

**Fig. 1:**
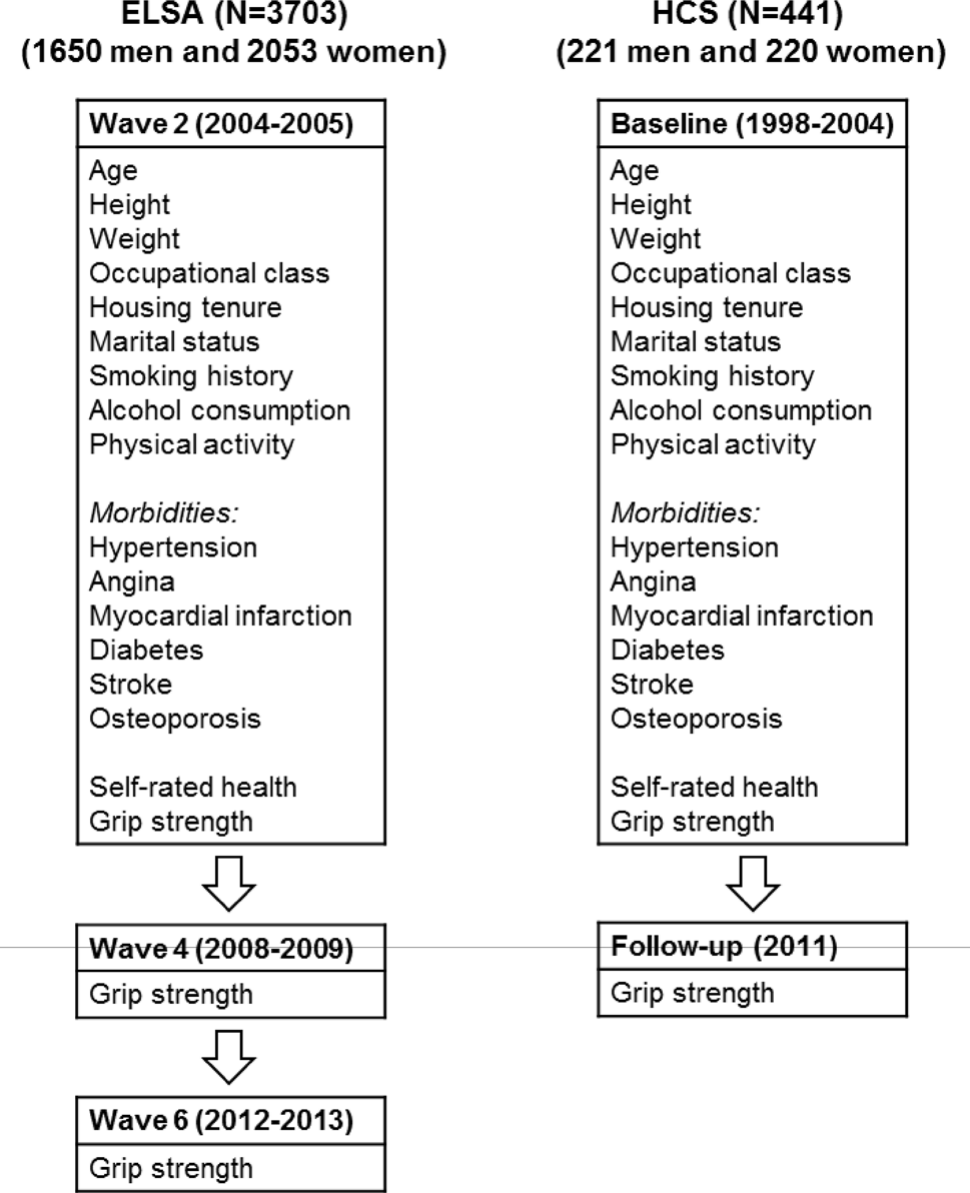
Phases of data collection for the English Longitudinal Study of Ageing and the Hertfordshire Cohort Study. Sample sizes are shown for individuals with complete grip strength data from baseline to the end of follow-up. Only participant characteristics used for this analysis are detailed. Full descriptions of the cohorts have been described previously [26, 27]. *ELSA* English Longitudinal Study of Ageing, *HCS* Hertfordshire Cohort Study

An overview of the waves of data collection and the participant characteristics relevant to this analysis are provided in Fig. 1. Variables were coded for analysis as detailed in Online Appendix 1. Sex-specific standard deviation (SD) scores for change in grip strength were calculated internally for each cohort. Grip strength change in ELSA was characterised by fitting sex-specific linear mixed-effects models with random intercepts and slopes for grip strength over the three time points. Sex-specific standard deviation scores for the random slopes were used as the measure of grip strength change. This measure of change was weakly correlated with baseline grip strength among men (*r* = − 0.081) and women (*r* = 0.002).

In HCS, change in grip strength was characterised by estimating sex-specific linear regression models for grip strength at follow-up on grip strength at baseline with adjustment for individual follow-up duration; standardised residuals from these models function as Twisk’s recommended measure of “residual change” [32] in grip strength over time when data from only two time points are available and yield a measure of change which is independent of baseline level.

Data were described using summary statistics. Linear regression was used to explore sex- and age-adjusted associations between baseline participant characteristics and both baseline grip strength level and change in grip strength in ELSA, with replication analyses in HCS. All statistically significant (*p* < 0.05) sex- and age-adjusted correlates of grip strength level or change were included in final mutually adjusted models in ELSA; for consistency, the same final mutually adjusted models for grip strength level and change were estimated in HCS. We checked that no additional HCS characteristics were significant (*p* < 0.05) if added to the final models for grip strength level or change as motivated by analysis of the ELSA dataset. Baseline grip strength was not included as a covariate in analyses for grip strength change.

Standard deviation scores were coded for continuous characteristics. Whenever a marker of adiposity was associated with level or change in grip strength with *p* < 0.05 after adjustment for sex and age, both height and a weight-for-height residual were included in subsequent mutually adjusted analyses to reflect potential effects of stature and adiposity.

Formal tests for interactions, combined with visual inspection of results from sex-specific analyses illustrated that correlates of level and loss of grip strength were similar among men and women. Therefore, men and women were pooled for analyses which were conducted using Stata, release 13 (StataCorp, College Station, TX, USA).

## Results

### Descriptive Statistics

Characteristics of ELSA and HCS participants with grip strength data available at all time points are presented in Table 1. Mean (SD) age at baseline was 63.5 (7.5) years in ELSA and 64.9 (2.7) years in HCS. Median (inter-quartile range) follow-up time was 7.8 (7.7, 8.0) years in ELSA and 10.6 (10.1, 11.6) years in HCS. Men had higher average grip strength than women at baseline in ELSA and in HCS. In spite of marked heterogeneity in rates of change, average annualised declines in grip strength were apparent among ELSA and HCS participants, with men experiencing accelerated rates of loss compared with women. Distributions of baseline level and annual change in grip strength among ELSA and HCS participants are illustrated in Online Appendix 2.

**Table 1:** Participant characteristics

*N* (%)	ELSA		HCS	
	Men (*n* = 1650)	Women (*n* = 2053)	Men (*n* = 221)	Women (*n* = 220)
Age at baseline (years)*	63.4 (7.5)	63.6 (7.5)	64.1 (2.5)	65.8 (2.7)
Height (cm)*	173.8 (6.8)	160.3 (6.3)	174.7 (6.5)	161.2 (5.9)
Weight (kg)*	84.4 (13.6)	72.2 (14.1)	81.2 (11.2)	70.0 (12.9)
BMI (kg/m^2^)*	27.9 (4.1)	28.1 (5.2)	26.6 (3.5)	26.9 (4.6)
Social class (manual)	597 (36.4)	827 (40.7)	121 (57.6)	120 (54.5)
Housing tenure (not owned/mortgaged)	176 (10.7)	289 (14.1)	26 (11.8)	34 (15.5)
Not currently married/cohabiting	280 (17.0)	697 (34.0)	29 (13.1)	53 (24.1)
Ever smoked	1145 (69.5)	1088 (53.0)	135 (61.1)	76 (34.5)
Alcohol consumer	1329 (86.5)	1371 (71.1)	194 (87.8)	124 (56.4)
Sedentary/Low physical activity	280 (17.0)	503 (24.5)	47 (21.3)	59 (26.8)
Morbidities				
Hypertension	637 (38.6)	802 (39.1)	60 (27.1)	72 (32.7)
Angina	160 (9.7)	123 (6.0)	10 (4.6)	12 (5.5)
Myocardial infarction	114 (6.9)	45 (2.2)	4 (1.8)	0 (0.0)
Diabetes	140 (8.5)	111 (5.4)	24 (10.9)	21 (9.7)
Stroke	51 (3.1)	60 (2.9)	7 (3.2)	9 (4.1)
Osteoporosis	27 (1.6)	174 (8.5)	7 (3.2)	27 (12.3)
Number of morbidities				
0	878 (53.2)	1073 (52.3)	133 (61.9)	110 (50.9)
1	510 (30.9)	724 (35.3)	57 (26.5)	79 (36.6)
2	184 (11.2)	189 (9.2)	22 (10.2)	22 (10.2)
3	62 (3.8)	58 (2.8)	3 (1.4)	5 (2.3)
4 or 5	16 (1.0)	9 (0.4)	0 (0.0)	0 (0.0)
Self-rated health				
Poor	66 (4.0)	83 (4.0)	2 (0.9)	1 (0.5)
Fair	233 (14.1)	352 (17.2)	6 (2.7)	20 (9.1)
Good	534 (32.4)	640 (31.2)	78 (35.3)	87 (39.5)
Very good	534 (32.4)	674 (32.8)	106 (48.0)	96 (43.6)
Excellent	283 (17.2)	303 (14.8)	29 (13.1)	16 (7.3)
Grip strength at baseline (kg)*	42.9 (8.7)	25.7 (5.9)	44.6 (7.0)	27.7 (5.0)
Grip strength at end of follow-up (kg)*	37.7 (8.9)	22.8 (6.0)	36.1 (7.4)	21.3 (6.0)
Annual change in grip (kg/year)*	− 0.66 (0.86)	− 0.38 (0.60)	− 0.74 (0.48)	− 0.64 (0.48)
Follow-up duration (years)^+^	7.8 (7.7, 8.0)	7.8 (7.7, 8.0)	11.6 (11.2, 11.9)	10.1 (9.7, 10.4)
Age at follow-up (years)*	71.2 (7.5)	71.4 (7.4)	75.6 (2.5)	75.8 (2.6)

Manual social class—HCS: categories IIIM, IV and V of SOC90, ELSA: ‘Manual’ or ‘Routine’ categories of NS-SEC

Alcohol consumer—HCS: drinking at least one unit per week, ELSA: drinking alcohol at least once per month

Low physical activity—HCS: Dallosso score ≤ 50, ELSA: low/sedentary

Osteoporosis HCS *t*-score < − 2.5 for femoral neck or lumber spine; ELSA: osteoporosis according to self-report

All summary statistics are for baseline phases of data collection except where indicated

*ELSA* English Longitudinal Study of Ageing, *HCS* Hertfordshire Cohort Study

*Mean (SD), + Median (lower quartile, upper quartile)

### Correlates of Level and Change in Grip Strength

Table 2 shows the associations between baseline characteristics and grip strength level and change in the two cohorts. In both ELSA and HCS, older age was associated with lower baseline level of grip strength and accelerated loss rate, and men had higher average baseline levels of grip strength than women. However, our principal derived estimates of change in grip strength were sex specific (see “Statistical Methods” section); this precluded detection of sex differences for change in grip strength.

**Table 2:** Sex- and age-adjusted associations between participant characteristics and grip strength level and change in ELSA and HCS

Participant characteristic	Grip strength level at baseline				Grip strength change^a^			
	Adjusted for sex and age		Mutually adjusted		Adjusted for sex and age		Mutually adjusted	
	Estimate (95% CI)	*p*	Estimate (95% CI)	*p*	Estimate (95% CI)	*p*	Estimate (95% CI)	*p*
Associations in ELSA								
Age (years)*	− 0.38 (− 0.41, − 0.35)	< 0.001	− 0.29 (− 0.32, − 0.26)	< 0.001	− 0.04 (− 0.04, − 0.03)	< 0.001	− 0.03 (− 0.04, − 0.03)	< 0.001
Sex (women)	− 17.16 (− 17.63, − 16.69)	< 0.001	− 16.87 (− 17.29, − 16.45)	<0.001	0.00 (− 0.06, 0.06)	1.000	0.03 (− 0.04, 0.09)	0.445
Height (*z*-score)*	1.86 (1.65, 2.07)	< 0.001	1.72 (1.50, 1.93)	< 0.001	0.06 (0.03, 0.10)	< 0.001	0.06 (0.03, 0.09)	< 0.001
Weight (*z*-score)*	1.31 (1.10, 1.52)	< 0.001			− 0.02 (− 0.05, 0.01)	0.177		
BMI (*z*-score)*	0.56 (0.35, 0.78)	< 0.001			− 0.05 (− 0.08, − 0.02)	0.002		
Weight-for-height residual (*z*-score)*	0.72 (0.51, 0.94)	< 0.001	1.02 (0.81, 1.24)	< 0.001	− 0.04 (− 0.07, − 0.01)	0.008	− 0.02 (− 0.06, 0.01)	0.170
Social class (manual)	− 1.08 (− 1.52, − 0.64)	< 0.001	− 0.41 (− 0.85, 0.03)	0.066	− 0.09 (− 0.15, − 0.02)	0.007	− 0.05 (− 0.12, 0.02)	0.133
Housing tenure (not owned/mortgaged)	−2.18 (−2.82,−1.53)	< 0.001	−0.96 (−1.62,−0.30)	0.005	−0.10 (−0.19,0.00)	0.040	−0.02 (−0.12,0.08)	0.674
Not currently married/cohabiting	− 0.96 (− 1.46, − 0.45)	< 0.001	− 0.38 (− 0.88, 0.12)	0.132	− 0.05 (− 0.12, 0.03)	0.223	− 0.02 (− 0.09, 0.06)	0.664
Ever smoked	0.09 (− 0.36, 0.54)	0.695			− 0.04 (− 0.11, 0.02)	0.192		
Alcohol consumer	0.40 (− 0.15, 0.94)	0.154			0.03 (− 0.05, 0.10)	0.523		
Low physical activity	− 1.99 (− 2.52, − 1.46)	< 0.001	− 1.45 (− 1.99, − 0.92)	< 0.001	− 0.11 (− 0.19, − 0.03)	0.005	− 0.06 (− 0.14, 0.02)	0.170
Self-rated health**	0.83 (0.63, 1.03)	< 0.001	0.61 (0.39, 0.83)	< 0.001	0.05 (0.03, 0.08)	< 0.001	0.03 (−0.01, 0.06)	0.098
Number of morbidities*	− 0.67 (− 0.93, − 0.41)	< 0.001	− 0.33 (− 0.60, − 0.06)	0.016	− 0.07 (− 0.11, − 0.04)	< 0.001	− 0.04 (− 0.08, 0.00)	0.046
Associations in HCS								
Age (years)*	− 0.49 (− 0.71, − 0.27)	< 0.001	− 0.37 (− 0.59, − 0.16)	0.001	− 0.06 (− 0.09, − 0.02)	0.001	− 0.05 (− 0.08, − 0.01)	0.015
Sex (women)	− 16.95 (− 18.09, − 15.81)	< 0.001	− 15.87 (− 17.01, − 14.72)	< 0.001	0.00 (− 0.19,0.19)	0.990	0.11 (− 0.09, 0.31)	0.280
Height (*z*-score)*	2.01 (1.48, 2.54)	< 0.001	1.84 (1.29, 2.38)	< 0.001	0.02 (− 0.07, 0.12)	0.616	0.01 (− 0.09, 0.10)	0.862
Weight (*z*-score)*	1.00 (0.45, 1.55)	< 0.001			− 0.06 (− 0.15, 0.03)	0.198		
BMI (*z*-score)*	− 0.02 (− 0.58, 0.54)	0.948			− 0.07 (− 0.16, 0.02)	0.147		
Weight-for-height residual (*z*-score)*	0.34 (− 0.22, 0.90)	0.232	0.49 (− 0.07, 1.04)	0.087	− 0.08 (− 0.17, 0.02)	0.107	− 0.02 (− 0.12, 0.08)	0.670
Social class (manual)	− 1.12 (− 2.25, 0.02)	0.054	− 0.57 (− 1.68, 0.54)	0.313	− 0.12 (− 0.31,0.07)	0.214	− 0.06 (− 0.25,0.14)	0.553
Housing tenure (not owned/mortgaged)	0.11 (− 1.53, 1.75)	0.895	0.76 (− 0.87, 2.40)	0.359	− 0.17 (− 0.44, 0.10)	0.223	− 0.10 (− 0.39, 0.18)	0.480
Not currently married/cohabiting	1.12 (− 0.33, 2.58)	0.130	0.89 (− 0.53, 2.31)	0.217	− 0.13 (− 0.37, 0.11)	0.300	− 0.10 (− 0.35, 0.15)	0.433
Ever smoked	− 1.19 (− 2.35, − 0.03)	0.044			− 0.20 (− 0.39, − 0.01)	0.040		
Alcohol consumer	1.17 (− 0.16, 2.49)	0.085			0.12 (− 0.10, 0.34)	0.278		
Low physical activity	− 0.64 (− 1.96, 0.67)	0.336	− 0.41 (− 1.71, 0.89)	0.534	− 0.03 (− 0.25, 0.19)	0.778	0.03 (− 0.19, 0.26)	0.767
Self-rated health**	0.99 (0.27, 1.71)	0.007	0.86 (0.13, 1.59)	0.021	0.17 (0.05, 0.29)	0.006	0.10 (− 0.03, 0.23)	0.118
Number of morbidities*	− 0.81 (− 1.58, − 0.03)	0.041	− 0.68 (− 1.45, 0.08)	0.079	− 0.14 (− 0.26, − 0.01)	0.036	− 0.11 (− 0.25, 0.02)	0.105

A positive regression coefficient for grip change illustrates that an increase/presence of the predictor was associated with reduced loss of grip strength over time and a negative coefficient reflects accelerated loss of grip strength

*ELSA* English Longitudinal Study of Ageing, *HCS* Hertfordshire Cohort Study, *p p* value

^a^Conditional change in grip strength was used as the outcome variable in HCS; in ELSA, change in grip was obtained using a residual multilevel-modelling approach

*Estimate per unit increase in characteristic

**Estimate per higher band of characteristic; Remaining estimates are for the presence versus absence of the characteristic

### Results from ELSA

**Fig. 2:**
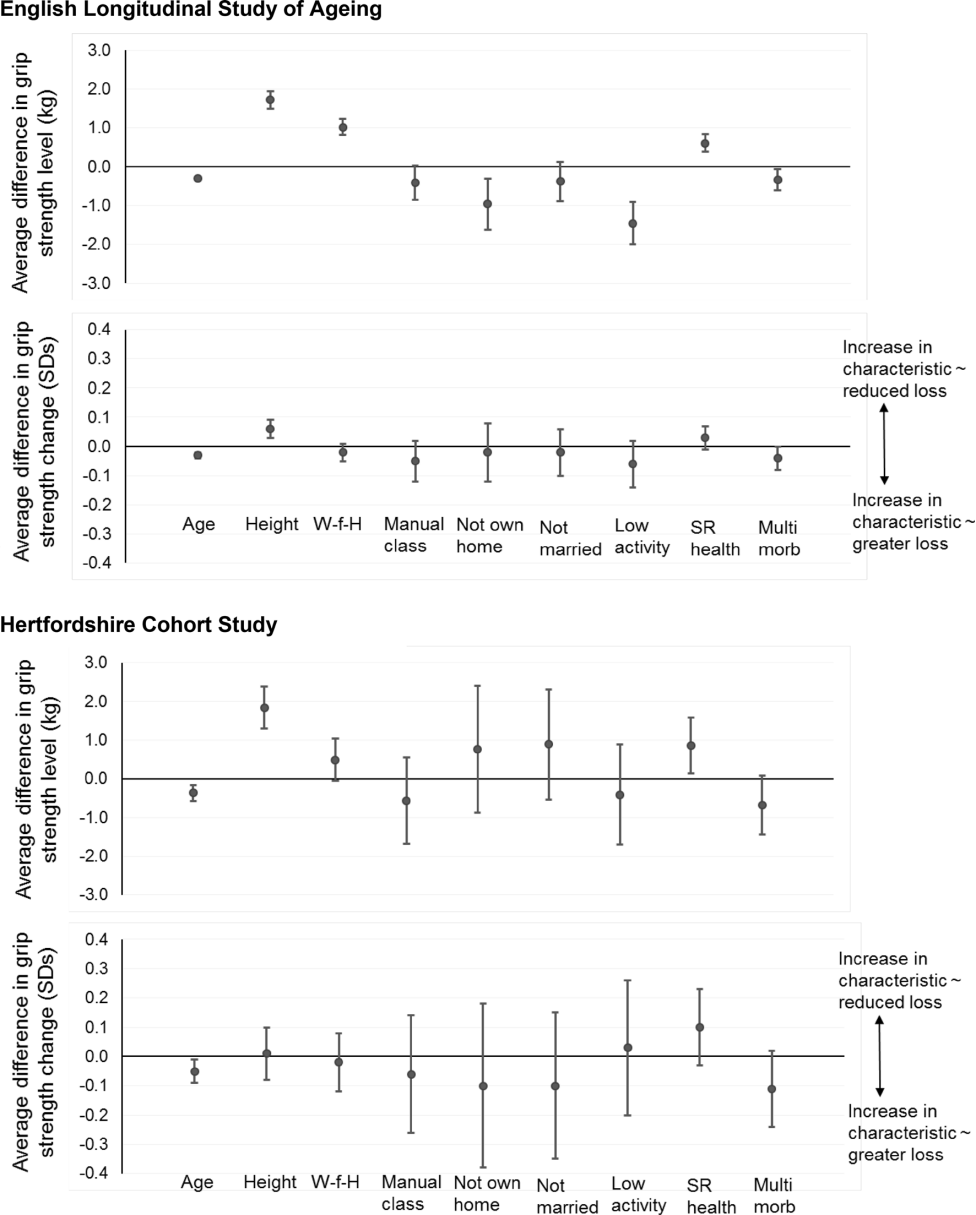
Mutually adjusted associations between participant characteristics and grip strength level and change (pooled and gender-adjusted). Estimates are per unit increase in age (years) and number of morbidities, per SD increase in anthropometry and per higher band of self-rated health. Estimates for the presence vs absence of the characteristics are shown for the remaining predictors. *W-f-H* weight-for-height residual, *SR* self-rated, *Multi morb* number of morbidities

In ELSA, the following characteristics were associated with lower grip strength level at baseline after adjustment for age and sex: shorter height, lower weight, BMI and weight-for-height residual (reduced adiposity); manual social class; not owner-occupying one’s home; not being currently married/cohabiting; lower physical activity; poorer self-rated health; and increased multimorbidity. Apart from marital status and occupational class, all these associations were significant in mutually adjusted analyses (Table 2; Fig. 2).

Accelerated loss of grip strength was associated with the following characteristics after adjustment for age and sex: shorter height; higher BMI and weight-for-height residual (increased adiposity); manual social class; not owner-occupying one’s home; lower physical activity; poorer self-rated health; and increased multimorbidity. However, only height and multimorbidity remained significant in mutually adjusted analyses (Table 2; Fig. 2).

### Results from HCS

A replication analysis in HCS identified similar correlates of grip strength level and loss to those identified in ELSA (Table 2; Fig. 2). However, associations in HCS were generally weaker in part owing to smaller sample size.

As in ELSA, the following characteristics were associated with lower grip strength level at HCS baseline after adjustment for age and sex: shorter height, lower weight, poorer self-rated health and increased multimorbidity. Smoking history was also associated with lower grip strength level in HCS. Only height and self-rated health were associated with grip strength level when the ELSA mutually adjusted model was estimated in HCS (Table 2; Fig. 2).

Similarly to ELSA, accelerated loss of grip strength in HCS was associated with poorer self-rated health and increased multimorbidity; however, these associations were not apparent when the ELSA mutually adjusted model for change in grip strength was estimated in HCS (Table 2; Fig. 2). Smoking history was also associated with accelerated loss of grip strength in HCS after adjustment for sex and age but this was not apparent when it was added to the mutually adjusted model.

## Discussion

We have used data from the English Longitudinal Study of Ageing and the Hertfordshire Cohort Study to examine correlates of level, and rate of loss, of grip strength in later life. Our results suggest that advancing age, shorter stature, and multimorbidity are correlates of both lower level and accelerated loss of grip strength in later life. Socioeconomic disadvantage, reduced adiposity, low level of physical activity and poorer self-reported health are important additional correlates of low grip strength level in later life, but play a weaker role as correlates of rate of loss of grip strength after adjustment for age, stature and multimorbidity.

Our work has some limitations. First, participant characteristics were not all measured according to identical protocols in ELSA and HCS and no comparable assessment of diet quality was available so we were not able to examine the relationship between diet and level and loss of grip strength; however, we reviewed the data dictionaries for the two studies and pragmatically harmonised data between them in the best way possible. Second, the age range of participants was wider in ELSA than HCS but the average duration of follow-up was somewhat longer in HCS than ELSA. Third, our assessment of grip strength change in ELSA was based on a multilevel random slopes and intercepts model for data measured over three waves of follow-up; our assessment of change in HCS was only based on two repeat measurements and derived using a residual change approach. Fourth, the ELSA sample size was much bigger than that available for HCS. Finally, different dynamometers were used in ELSA and HCS. However, a high correlation has been demonstrated between measurements made using these two devices [33]. Moreover, as each cohort analyses was internal, the fact that the studies used different devices should not have biassed our assessment of the determinants of level and change in grip strength. In spite of these various limitations, the results that we obtained about risk factors for level and loss of grip strength in later life were consistent in the two cohorts.

Our study also has many strengths. First, we harmonised data from two large, well-characterised, population-based cohorts in the United Kingdom. We regarded ELSA (which was designed to be representative of the community-dwelling population aged over fifty in England) as our principal analysis cohort and utilised HCS as a replication sample. Our conclusions about the important predictors of level and loss of grip strength in later life were strikingly consistent in the two cohorts, although associations in HCS were less statistically significant owing to smaller sample size. Second, we were careful to estimate change in grip strength using statistical techniques that were appropriate to the extent of information available in each cohort (three waves of follow-up for ELSA and two for HCS) and which each yielded a measure of change that was independent of initial level. Finally, we have considered a wide panel of potential determinants of level and loss of grip strength in later life.

To our knowledge, this novel study is the first to systematically examine whether level and loss of grip strength in later life share similar risk factors. Our findings that older age, shorter stature, multimorbidity, socioeconomic disadvantage, reduced adiposity, low level of physical activity and poorer self-reported health are risk factors for weaker grip strength in later life are consistent with an extensive published literature [5, 12, 14–17]. Published evidence pertaining to risk factors for accelerated loss of muscle strength in later life is more limited but is consistent with our conclusion that older age and multimorbidity are key risk factors for accelerated loss of grip strength in later life. For example, cross-sectional [5] and longitudinal studies [7] have clearly demonstrated that grip strength declines with advancing age, irrespective of health status [34], and a range of studies from the UK [35], Europe [22, 23] and North America [34] have shown that cardiovascular, endocrine and respiratory morbidity are associated with level and loss of grip strength in later life.

In a Swedish study of men and women aged 50–88 years, using data on risk factors measured up to 20 years before grip strength was assessed, there were marked differences between the sexes, such that stress, smoking and dementia were the only variables associated with grip strength decline in women, while chronic disease, lower physical activity at work, higher mean arterial pressure and being married were the only variables associated with decline in grip strength in men [22]. Further evidence that influences on the trajectory of grip strength may vary between the sexes came from a study of a cohort of people aged 85 and over in Newcastle, UK [25]. Of a range of risk factors examined, greater physical activity was the only factor significantly associated with slower decline in grip strength and in the sample as a whole, this association was only present in men. In a large cohort of Afro-Caribbean men, greater body mass index and lower lean mass were the only factors associated with rate of grip strength decline independently of lifestyle and medical history [24], while in a cohort of Finnish men and women, excess weight, smoking, chronic disease and lower physical activity in midlife were associated with decline over 22 years [23]. In this latter study, there was no evidence that determinants of decline in grip strength varied by sex. That is consistent with findings reported in the current study.

We are not aware of any studies that have identified shorter stature as a risk factor for accelerated loss of grip strength in later life but this was a striking finding in our analysis of data from ELSA. Adult height is a marker of cumulative lifetime nutrition (especially that experienced during early life), biological deprivation and standard of living [36]. Developmental influences on level of muscle strength in later life are also well recognised [37, 38] and have been implicated in the acquisition of muscle strength during childhood [39] and young adulthood [40]. Considered in this context, our current study suggests that developmental influences may also have an impact on rate of decline in muscle strength. However, in HCS, we found no association between birth weight (a marker of adverse foetal environment) and rate of loss of grip strength (data not shown).

In conclusion, we have shown that a host of anthropometric, socioeconomic, physical, psychosocial and medical factors are associated with grip strength level in older age. In contrast, only advancing age, shorter stature and multimorbidity are associated with subsequent accelerated rate of decline in muscle strength. These findings suggest that multimorbidity is an important modifiable determinant of loss of muscle strength in later life, and raise the possibility that developmental influences may have an impact on rate of involutional decline in muscle strength. These results will inform the development of lifecourse intervention strategies to promote maintenance, and reduce loss, of muscle strength in later life.

## Electronic supplementary material

Below is the link to the electronic supplementary material.

Supplementary material 1 (DOCX 669 kb)
