# Supplementary material for: Correlates of Level and Loss of Grip Strength in Later Life: Findings from the English Longitudinal Study of Ageing and the Hertfordshire Cohort Study
Source: Calcif Tissue Int. 2017 Oct 22;102(1):53–63. doi: 10.1007/s00223-017-0337-5 (PMC5760591; doi:10.1007/s00223-017-0337-5)
Supplement: Supplementary file 1 — Supplementary material 1 (DOCX 669 kb) [file 223_2017_337_MOESM1_ESM.docx]

**Article title:** Correlates of level and loss of grip strength in later life: findings from the English Longitudinal Study of Ageing and the Hertfordshire Cohort Study

**Journal:** Calcified Tissue International and Musculoskeletal Research

**Authors**: HE Syddall, LD Westbury, SC Shaw, EM Dennison, C Cooper (corresponding author), CR Gale

**Affiliations and e-mail address for corresponding author**:

MRC Lifecourse Epidemiology Unit, University of Southampton, Southampton, UK

NIHR Southampton Biomedical Research Centre, University of Southampton and University Hospital Southampton NHS Foundation Trust,

Southampton, UK

NIHR Musculoskeletal Biomedical Research Unit, University of Oxford, Oxford, UK

cc@mrc.soton.ac.uk

| **Appendix 1: Ascertainment and coding of variables from the English Longitudinal Study of Ageing and the Hertfordshire Cohort Study** | | | | | |
| --- | --- | --- | --- | --- | --- |
|  |  | | |  | |
|  | **Ascertainment** | | | **Coding for our analysis** | |
| ***Domain*** | **ELSA** | **HCS** | | **ELSA** | **HCS** |
|  |  |  | |  |  |
| ***Anthropometry*** |  |  | |  |  |
| Age | Self-reported | Verification of date of birth from Heath Visitors’ register | | Continuous variable | Continuous variable |
| Height | Measured using portable stadiometer at home interview | Measured using Harpenden pocket stadiometer at clinic | | Continuous variable | Continuous variable |
| Weight | Measured using electronic scales at home interview | Measured using SECA floor scale at clinic | | Continuous variable | Continuous variable |
| Weight adjusted for height | Derived | Derived | | To avoid multicollinearity problems owing to correlation between measurements of height and weight, for each cohort a sex-specific standardised residual of weight-adjusted-for-height was calculated for inclusion with height in regression models. | |
| BMI (kg/m^2^) | Derived | Derived | | Derived: (weight kg)/(height in metres, squared) | Derived: (weight kg)/(height in metres, squared) |
|  |  |  | |  |  |
| ***Lifestyle*** |  |  | |  |  |
| Smoking history | Self-reported | | | Dichotomised to ever vs never smoked regularly | |
| Alcohol intake | Self-reported | | | Dichotomised to ‘drinkers (consuming alcohol at least once per month)’ and ‘non-drinkers’ | Dichotomised to ‘drinkers (≥1 unit per week)’ and ‘non-drinkers’ |
| Activity score | Self-reported physical activity in work and in daily life | Self-reported using Dallosso customary physical activity questionnaire^1^ | | A categorical variable was derived (sedentary, low, moderate or high) that approximates closely to the classification used in the Allied Dunbar Survey of Fitness; we dichotomised this into (sedentary/low vs moderate/high)^7^ | Low physical activity defined as Dallosso score ≤50 |
|  |  |  | |  |  |
| ***Social Circumstances*** |  |  | |  |  |
| Social class | Self-reported occupation(s) coded to National Statistics Socio-economic Classification^2^ | Self-reported occupation(s), coded to 1990 Standard Occupational Classification^3^ using computer assisted standard occupational coding^4^ | | Dichotomised to ‘manual’ vs ‘non-manual’ | Dichotomised to ‘manual’ vs ‘non-manual’ on the basis of own current or most recent occupation , or that of the husband for ever-married women. |
| Relationship status | Self-reported | | | Dichotomised to ‘currently married/cohabiting’ vs ‘not’ | |
| Home ownership | Self-reported | | | Dichotomised to ‘own/mortgage home’ vs ‘not’ | |
|  |  | |  |  |  |
| Self-rated health: | Five point scale | | Five point scale within the SF-36 questionnaire^5^ | Ordinal variable | Ordinal variable |
|  |  | |  |  |  |
| ***Morbidities*** |  | |  |  |  |
| Angina | Self-reported previous diagnosis by doctor | | Self-reported typical angina via Rose chest pain questionnaire^6^ | Binary variable | Binary variable |
| Stroke | Self-reported previous diagnosis by doctor | | Self-reported previous diagnosis of stroke or transient ischaemic attack by doctor | Binary variable | Binary variable |
| Myocardial Infarction (MI) | Self-reported previous diagnosis by doctor | | Self-reported previous diagnosis by doctor | Binary variable | Binary variable |
| Osteoporosis (OP) | Self-reported previous diagnosis by doctor | | DXA scan for bone mineral density at lumber spine and proximal femur (Hologic QDR4500 machine) | Binary variable | Defined as a t-score of < -2.5 for either lumber spine or total femoral neck BMD. Binary variable. |
| Hypertension (HTN) | Self-reported previous diagnosis by doctor | | Self-reported use of antihypertensive medication and blood pressure measured three times after resting at clinic using Dinamap model 8101. | Binary variable | Hypertension defined as average measured BP ≥160/100 mmHg or medication known to affect blood pressure. Binary variable. |
| Diabetes (DM) | Self-reported previous diagnosis by doctor | | Self-reported previous diagnosis by doctor and method of control. Fasting 2 hour OGTT conducted at clinic for people without known diabetes | Binary variable | Diabetes defined as 2h plasma glucose ≥11.1mmol/l (WHO criteria^8^) or self-reported diagnosis of diabetes with treatment by diet control, tablets or insulin injections. Binary variable. |
|  |  | |  |  |  |
| Number of morbidities | Derived | | | Sum of binary variables detailing angina, MI, OP, HTN and DM. | Sum of binary variables detailing angina, MI, OP, HTN and DM. |
|  |  |  | |  |  |

Self-reported measures were ascertained by nurse administered home interview in ELSA and HCS.

***References to Appendix 1:***

1. *Dallosso* HM, Morgan K, Bassey EJ, Ebrahim SBJ, Fentem PH, Arie THD. Levels of customary physical activity among the old and the very old living at home. Journal of *Epidemiology and Community Health. 1988;42:121-7*.
2. *Atherton K, Power C. Health inequalities with the National Statistics-Socioeconomic classification: disease risk factors and health in the 1958 British birth cohort. European Journal of Public Health 2007;****17****(5):486-91.*
3. *Office of Population Censuses and Surveys. Standard occupational classification, Vol 1 Structure and definition of major, minor and unit groups. London: HMSO, 1990.*
4. *Elias P, Halstead K, Prandy K. Computer Assisted Standard Occupational Coding: H.M. Stationery Office; 1993.*
5. *Ware JE, Kosinski M, Gandek B. SF-36 Health Survey: Manual and Interpretation Guide. Lincoln, RI: Quality Metric Incorporated, 2000.*
6. [*Rose GA*](https://www.ncbi.nlm.nih.gov/pubmed/?term=Rose%20GA%5BAuthor%5D&cauthor=true&cauthor_uid=4972212)*,*[*Blackburn H*](https://www.ncbi.nlm.nih.gov/pubmed/?term=Blackburn%20H%5BAuthor%5D&cauthor=true&cauthor_uid=4972212)*. Cardiovascular survey methods.* [*Monogr Ser World Health Organ.*](https://www.ncbi.nlm.nih.gov/pubmed/4972212)*1968;56:1-188.*
7. *Activity and Health Research. Allied Dunbar National Fitness Survey. A report on Activity Patterns and Fitness levels: Main Findings. London: Sports Council and Health Education Authority, 1992.*
8. *World Health Organisation. Definition, diagnosis and classification of diabetes mellitus and its complications. Part 1: Diagnosis and classification of diabetes mellitus. Geneva, 1999.*

**Appendix 2: Distributions of baseline level and change in grip strength among participants in the English Longitudinal Study of Ageing and the Hertfordshire Cohort Study**


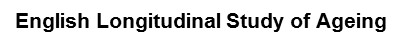

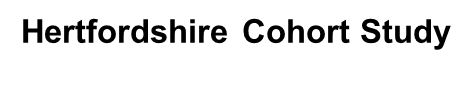

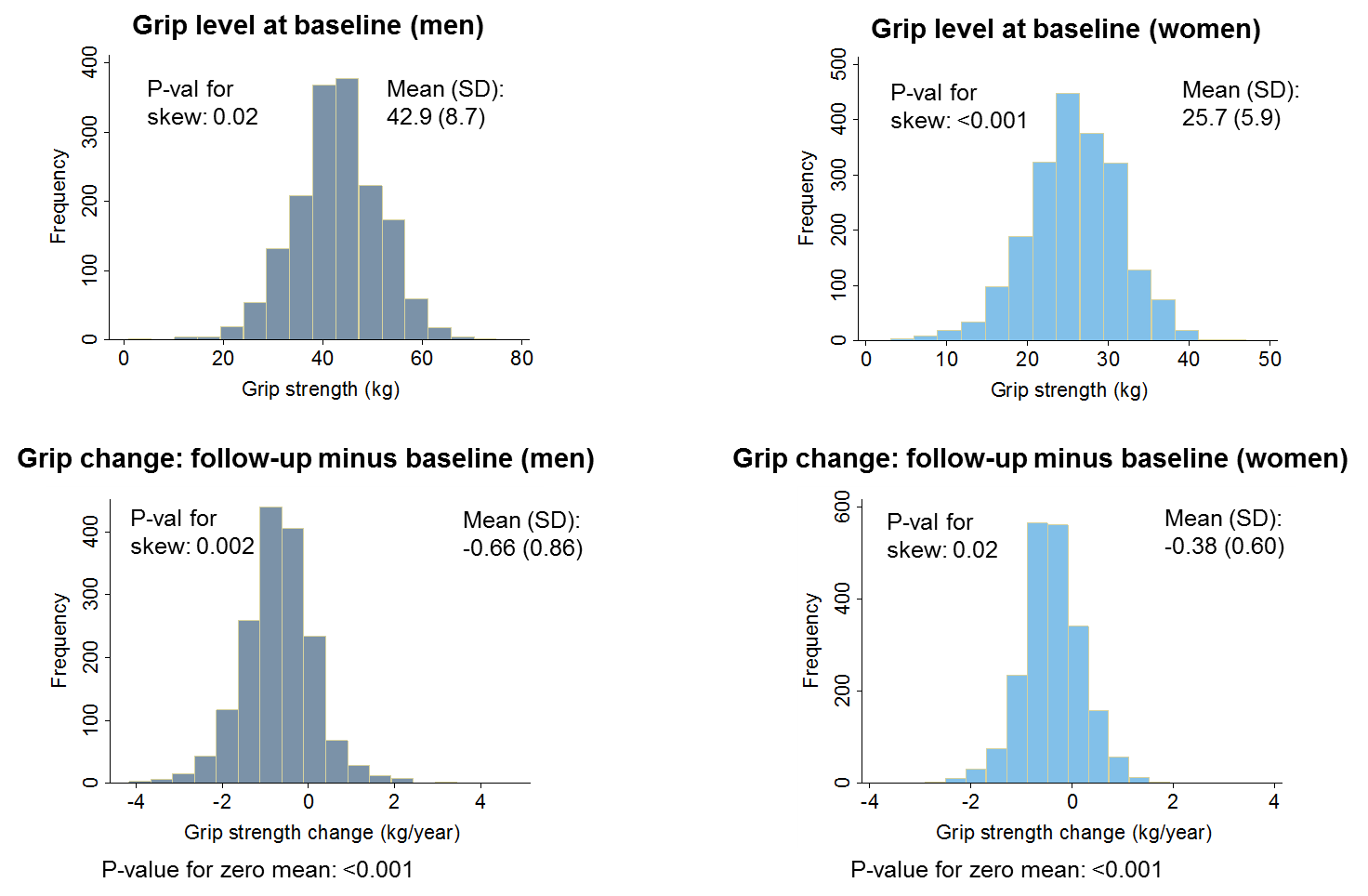

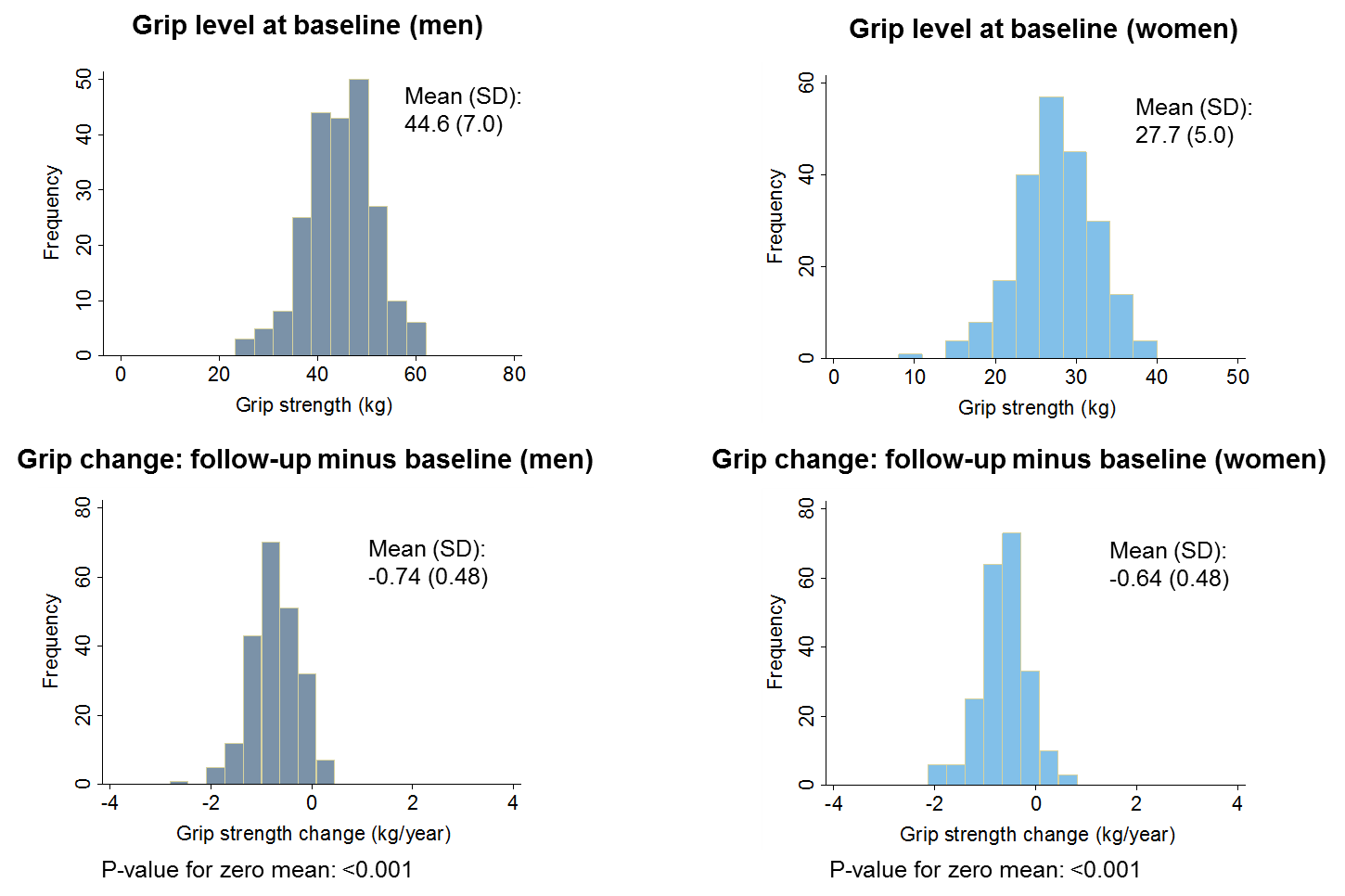


Crude annualised changes in grip strength ([follow-up -baseline]/follow-up duration) are shown for illustrative purposes but conditional assessments of change, as detailed in the Statistical Methods section were used in all other analyses.

P-values for a zero difference in mean grip strength change over time were calculated from t-tests.

*Distributions of level and change in grip strength in ELSA and HCS*

The frequency distributions of grip strength level at baseline, and crude annualised change in grip strength (calculated as final follow-up grip strength minus baseline grip strength, divided by follow-up duration), among ELSA and HCS participants are shown in Appendix 2. Graphical inspection did not identify marked skewness in the distributions of level or change in grip strength among men or women in either cohort. However, there was substantial variation between individuals both in terms of grip strength level at baseline and change in grip strength over time.
